# The prevalence of and the effect of global stressors on eating disorders among medical students

**DOI:** 10.3389/fpsyg.2025.1507910

**Published:** 2025-02-04

**Authors:** Muneera B. Almahmeed, Muna A. Almutawa, Yahya M. Naguib

**Affiliations:** ^1^College of Medicine and Health Sciences, Arabian Gulf University, Manama, Bahrain; ^2^Physiology Department, College of Medicine and Health Sciences, Arabian Gulf University, Manama, Bahrain; ^3^Clinical Physiology Department, Faculty of Medicine, Menoufia University, Menoufia, Egypt

**Keywords:** mental health, behavior, stress, eating disorders, medical students, global stressors

## Abstract

**Background:**

Eating disorders (EDs) are among the most serious forms of psychiatric illness, potentially leading to multi-systemic complications. Owing to their high stress levels, medical students are more likely to experience mental health difficulties that predispose them to developing EDs. Societal stigmatization and misinformation, especially in a middle eastern context, contribute to the underreporting and challenges in seeking the appropriate treatment at an early stage, increasing the risk of complications and mortality. EDs remain an under-studied phenomena in the middle east, limiting understanding and treatment options.

**Objective:**

The objectives of the current study were to assess the prevalence of EDs, potential contributing factors, and the impact of the COVID-19 pandemic as an independent global stressor among medical students at the Arabian Gulf University.

**Methods:**

This cross-sectional study consisted of a three-part self-administered questionnaire comprising of demographic data, the Eating Attitudes-26 (EAT-26) test, and COVID-19 associated stressors. The study was conducted on medical students at the AGU in the preparatory year up to Year 6. Data was collected from September 2022 to February 2023.

**Results:**

Three hundred and ninety-seven students were recruited in the present study. 32.1% of the tested students demonstrated an elevated risk for developing EDs. Living situation, earlier years of study, and mental health status were significantly associated with increased risk. Response to global stressors such as COVID-19 pandemic was significantly correlated to worsening EDs status in high-risk individuals.

**Conclusion:**

Eating disorders have been discovered to be highly prevalent among medical students, which brings to light an undervalued health concern. This warrants both awareness programs on campus, and the development of individual-tailored eating disorder treatment programs to halt progression and increase overall quality of life and education.

## Introduction

Eating disorders (EDs) encompass a group of complex psychiatric syndromes that can cause a multitude of serious health and psychosocial complications (Balasundaram and Santhanam, 2024; Phillips et al., 2015). According to the Diagnostic and Statistical Manual of Mental Disorders Fifth Edition (DSM-V), EDs are subcategorized mainly as Anorexia Nervosa (AN), Bulimia Nervosa (BN), Pica, Rumination disorder, Avoidant/restrictive food intake disorder (ARFID), Binge-eating disorder (BED), Other Specified Feeding or Eating Disorder (OSFED) (Call et al., 2013). Anorexia Nervosa, the most well-documented ED so far, is the restrictive form where food consumption is significantly limited, whereas Bulimia Nervosa is characterized by frequent episodes of uncontrolled binge eating followed by compensatory behaviors such as self-induced vomiting, laxative/diuretic usage, fasting or vigorous exercise (Golden et al., 2003; Nitsch et al., 2021; Walsh, 2013). ARFID, previously known as Eating Disorder Not Otherwise Specified (EDNOS) is a heterogenous group of eating disorders that includes purging disorder, binge eating disorder (BED) and aspects of both AN and BN, where patients typically do not meet any straightforward criteria (Smink et al., 2012). A significant number of young individuals have indicated that they have suffered from an eating disorder. It has been reported that 5.5–17.9% of young women and by 0.6–2.4% of young men have experienced a DSM-5 eating disorder before reaching early adulthood (Silén and Keski-Rahkonen, 2022).

Students with EDs demonstrate a reduced ability to concentrate, irritability as well as physical symptoms such as lethargy, nausea, and headaches which cause decreased academic performance and motivation (Ogundele, 2018). The physical complications of EDs are vast, leaving no organ system unaffected. Some clinical complications caused by EDs include electrolyte disturbances, endocrine, cardiac, hematologic, neurological, and renal complications, which can be severe and potentially irreversible when not identified and treated early (Cartwright, 2004; Messerli-Bürgy et al., 2010). Indeed, early recognition of mild eating disorders can curb the progression of disordered eating attitudes and behaviors to more serious forms of ED, preventing physical and psychological deterioration (Golden, 2003; Pinhas et al., 2011).

According to a systematic review that examined data from 2000 to 2018, the point prevalence of eating disorders is 5.7% in women and 2.2% in men (Galmiche et al., 2019). Medical students constitute a particularly vulnerable population to developing eating disorders owing to chronic stress, elevated rates of depression, anxiety, and risk of burnout (Dyrbye et al., 2006; Puthran et al., 2016). Expectedly, the global estimate among medical students is much higher than this, estimated to be 10.4% as documented by a recent systematic review and meta-analysis (Jahrami et al., 2019). This preexisting vulnerability has been exacerbated by the COVID-19 pandemic in multiple ways, of which cause widespread fear, disruption to academic learning and social life (Tavolacci et al., 2021). According to a study done during the pandemic, the chief source of stress was academic performance, uncertainty regarding the pandemic, financial insecurity, and health concerns (Salazar-Fernández et al., 2021; Wang et al., 2020). Moreover, students’ levels of anxiety, loneliness, and depression worsened, relative to measures before the crisis (Elmer et al., 2020). This is incredibly significant as it has been described that emotional distress is a mediator of coping behaviors such as eating comfort food (Salazar-Fernández et al., 2021).

Although the topic of eating disorders is a well-established issue in the general populace, particular groups like medical students, notably in the Arab region, face increased vulnerability because of academic pressures (Abdulla et al., 2023). The etiology of EDs is multifaceted, including biological, psychological, developmental, and socio-cultural factors (Pedersen et al., 2018). The latter factor is of particular interest in the Arab region, due to the rapid socio-cultural changes, western influence, widespread media use which have caused a preference for a thinner body type (Melisse et al., 2020). Means to a sedentary lifestyle are also more apparent in the Arab region, due to a marked utilization of mobile vehicles as a means of transport, access to cheap migrant labor especially domestically, high temperatures deterring outdoor activities and widespread of western style fast food. Furthermore, food is a crucial part of socialization in Arab societies, where large amounts of high-fat and high-carbohydrate foods are typically consumed. This practice, considering the restriction in opportunity for physical activity, breeds frustration around body weight and perception (Melisse et al., 2020; Badran and Laher, 2011). Unfortunately, the prevalence of the eating disorders in Arab medical students remains understudied. A study investigating eating disorders in medical students has been carried out in Lebanon, while few other studies have been conducted in Egypt, and Saudi Arabia investigating university students in general (Abo Ali, 2020; Bizri et al., 2020; El-Akabawy et al., 2022). Several studies concluded that there seemed to be a high proportion of students that were considered at high risk for eating disorders. This finding necessitates further studies into this topic to verify the findings to be true in other areas of the Arab world. Additionally, the COVID-19 pandemic has created a global environment which could exacerbate ED risk and symptoms. The widespread disruption to daily routines and lifestyles, decreased access to medical and psychological care and general insecurity regarding the future could worsen ED symptoms or perhaps create a setting favoring EDs (Willroth et al., 2023). The trend of eating disorders during the COVID-19 pandemic among university students has been studied in Saudi Arabia, which uncovered that 31.5% of female participants had a high risk of developing eating disorders (El-Akabawy et al., 2022).

Generally, the effects of the COVID-19 pandemic on medical students in the Arab region, particularly in Bahrain, has not been sufficiently investigated. This study aims to investigate the prevalence of eating disorder patterns among students in the Arabian Gulf university and to uncover any existing risk factors. Further objectives include discovering the effect of the COVID-19 pandemic in exacerbating existing EDs and to observe a possible association between demographic data and EDs risk. This is likely one of the first studies that investigates the prevalence of disordered eating behaviors in a Bahraini Medical School during the COVID-19 pandemic.

## Subjects and methods

A cross-sectional study was conducted at the College of Medicine and Health Sciences (CMHS) -Arabian Gulf University (AGU) in The Kingdom of Bahrain between September 2022 and March 2023.

### Ethical considerations

All the work and research procedures were carried out in compliance with the rules and recommendations of the Research and Ethics Committee at the AGU. Participants’ anonymities were preserved, and their right to refuse participation was respected. The ethical approval for this study was granted by the Research and Ethics Committee of the College of Medicine and Health Sciences at Arabian Gulf University (E29-PI-6-22). Informed consents were obtained from the participants either electronically or in person.

### Enrolment and survey distribution

Adopting a systematic random sampling technique, all the 1,236 medical students enrolled at the CMHS were eligible to participate in this study, from foundation year to sixth year medical students. This ensured that samples are chosen strictly by chance, provided equal odds for every member of the population to be chosen as a participant in this study, avoided sample bias. A link to an electronic version of the Eating attitude test-26 (EAT-26) was sent to all medical students of AGU. The questionnaire was further disseminated via social networking platforms. To further enhance the response rate, in-person interactions with the students were conducted to increase their awareness and encourage their participation in the study. Throughout the procedure, there were no duplicate responses per participant. Among the 1,236 medical students, 397 responses were obtained.

### Survey components

The survey consisted of general questions related to students’ demographic data, as well as a questionnaire that screened for eating disorders’ risk: Eating attitude test-26 (EAT-26). The EAT-26 has been reproduced with permission (Garner et al., 1982). Additional questions were included to explore recent familial or social stressors, the use of mental health services, current year of medical education and living arrangements. Finally, the questionnaire addressed the effect of COVID-19 pandemic on the status of EDs.

### EAT-26

The EAT-26 is a frequently used standardized self-report measure for eating disorder symptoms and concerns. It is a revised version of the EAT-40, which was developed in 1979 and has been extensively used in research to explore socio-cultural influences on eating disorder development and maintenance. The EAT-26 has been translated into numerous languages and has been employed in numerous studies. The EAT-26 is a valuable tool for evaluating the potential risk of developing eating disorders in groups such as high school and college students, as well as athletes. The underlying principle of using screening methods for eating disorders is that early detection can facilitate early intervention, which can help to minimize severe physical and mental complications, and even prevent fatalities. The recommended approach is to use the EAT-26 as a preliminary screening method, followed by an interview with a trained professional for individuals who score 20 or higher on the test (Papini et al., 2022).

### Measurements

The Eating attitude test-26 (EAT-26) is a 3-section self-administered questionnaire that is used to screen for eating disorders risk. Section A represents the socio-demographic data questions. Section B questions the behavioral weight-control patterns that includes self-reported binge eating, self-induced vomiting, laxative diet pills or diuretics use, excessive exercising to control weight. Section C consists of the same behavioral patterns in the past 6 months, such as a drastic weight loss of more than 8 kg over the past 6 months. The scoring for sections A, B and C is based on a 6-point scale system ranging from always (6) to never (0). However, the concluding question was scored inversely. The total sum of EAT-26 scores ranges from 0 to 78. Interpretation of scores is standardized as per protocol to identify high-risk individuals. Individuals scoring 20 or above were classified in the high-risk group.

### Statistical analysis

Data were analyzed using the Statistical Package for Social Sciences (SPSS), version 29 (IBM Corp., Armonk, NY, United States), and a *p*-value <0.05 was considered statistically. The Shapiro–Wilk normality test was performed to evaluate the normal distribution of the data. Categorical variables were signified as frequencies and percentages. Fisher’s Exact test was used to determine associations between different characteristics and outcomes.

## Results

In the present study, 397 medical students were recruited. Gender distribution thoroughly reflected the raw demographic data of the university, the majority being female respondents (75.3%). Most participants were in their preclinical years; years 1–4. Of concern, 68.8% of the sample reported experiencing mental health issues, yet only 18.6% sought professional help (Table 1).

**Table 1:** Sociodemographic criteria of the studied students.

	The studied students (*N* = 397)
Age	
17–20	235 (59.2)
20–24	145 (36.5)
>24	17 (4.3)
Sex	
Male	98 (24.7)
Female	199 (75.3)
Year of study	
Foundation year	17 (4.3)
Grade 1	70 (17.6)
Grade 2	92 (23.2)
Grade 3	72 (18.1)
Grade 4	47 (11.8)
Grade 5	42 (10.6)
Grade 6	57 (14.4)
Nationality	
Bahraini	124 (31.2)
Saudi	62 (15.6)
Kuwaiti	159 (40.1)
Omani	49 (12.3)
Lebanese	1 (0.3)
Sudanese	1 (0.3)
UAE	1 (0.3)
Marital status	
Single	391 (98.5)
Married	6 (1.5)
Living situation	
With family	152 (38.3)
Private shared apartment	37 (9.3)
University dorms	75 (18.9)
Alone	133 (33.5)
Are you currently seeking mental health support?	
Yes	74 (18.6)
No	323 (81.4)
Presence of recent stressors (academic pressure, financial difficulties, family issues, social issues etc.):	
Yes	273 (68.8)
No	124 (31.2)

Our findings revealed that 32.1% of participants were categorized as being at a higher risk for developing an eating disorder (Table 2). An evident association was concluded in the results between the risk of developing an eating disorder and the study year. Notably, students in clinical years exhibited a lower risk compared to those in preclinical years. Additionally, the students’ living situation was found to be significantly relative to the risk of developing an eating disorder. Participants living alone or with their families had the highest risk, while those living in private shared apartments and university dorms exhibited a lower risk. The results also showed non-local students constituted a proportion (63.9%) of the high-risk group. No significant associations were found between age, gender, nationality, marital status, and an increased risk of developing EDs (Table 3).

**Table 2:** Prevalence of EDs among medical students.

EDs	The studied students (*N* = 397)
Low risk	269 (67.9)
High risk	128 (32.1)

EDs, eating disorders.

**Table 3:** Sociodemographic criteria in relation to the high risk of EDs among the studied students.

	The studied students *N* = 397		Test	*p*-value
	Low risk *N* = 269	High risk *N* = 128	FE	
Age				
17–20	159 (59.1)	76 (59.4)	0.72	0.70
20–24	100 (37.2)	45 (35.2)		
>24	10 (3.7)	7 (5.5)		
Sex				
Male	69 (25.7)	29 (22.7)	0.42	0.52
Female	200 (74.3)	99 (77.3)		
Year of study				
Foundation year	7 (2.6)	10 (7.8)		
Grade 1	53 (19.7)	17 (13.3)		
Grade 2	65 (24.2)	27 (21.1)	12.45	0.05*
Grade 3	41 (15.2)	31 (24.2)		
Grade 4	32 (11.9)	15 (11.7)		
Grade 5	30 (11.2)	12 (9.4)		
Grade 6	41 (15.2)	16 (12.5)		
Nationality				
Bahraini	86 (32.0)	38 (29.7)		
Saudi	43 (16.0)	19 (14.8)		
Kuwaiti	97 (36.1)	62 (48.4)	9.28	0.16
Omani	40 (19.4)	9 (0.7)		
Lebanese	1 (0.4)	0 (0.0)		
Sudanese	1 (0.4)	0 (0.0)		
UAE	1 (0.4)	0 (0.0)		
Marital status				
Single	265 (98.5)	126 (98.4)	0.003	1.0
Married	4 (1.5)	2 (1.6)		
Living situation				
With family	101 (37.5)	51 (39.8)		
Private shared apartment	25 (9.3)	12 (9.4)	9.72	0.03*
University dorms	61 (22.7)	14 (10.9)		
Alone	82 (30.5)	51 (39.8)		
Currently seeking mental health support			9.44	0.002*
Yes	39 (14.5)	35 (27.3)		
No	230 (85.5)	93 (72.7)		
Presence of recent stressors (academic pressure, financial difficulties, family issues, social issues etc.).			1.33	0.25
Yes	180 (66.9)	93 (72.7)		
No	89 (33.1)	35 (27.3)		

EDs, eating disorders, ^*^significant; FE, Fisher’s Exact test.

In this study, 43.8% of the participants reported a deterioration in their eating habits during the COVID-19 pandemic. Furthermore, 44.1% expressed dissatisfaction with their food habits during the pandemic. Interestingly, 51.1% revealed an increased obsession with their weight and body image during the pandemic. There was a significant association between the response to COVID-19 as a global stressor and subjects at high risk for developing EDs (Table 4).

**Table 4:** Relation between COVID pandemic as a global stressor and the risk of EDs.

	The studied students (*N* = 397)			Test	*P*-value
	Low risk *N* = 269	High risk *N* = 128	Total	FE	
COVID-19 pandemic significantly worsened eating behavior					
Yes	102 (37.9)	72 (56.3)	174 (43.8)	14.04	<0.001*
No	167 (62.1)	56 (43.7)	223 (56.2)		
Are you more satisfied with your food habits prior to the COVID-19 pandemic?				5.92	0.05*
Yes	116 (43.1)	59 (46.1)	175 (44.1)		
No	153 (56.9)	69 (53.9)	222 (57.9)		
Did you regularly use food as a means of comfort during the pandemic?				3.57	0.06
Yes	141 (52.4)	80 (62.5)	221 (55.7)		
No	128 (47.6)	48 (37.5)	176 (44.3)		
The pandemic has made me more obsessed with my weight and body image.				52.17	<0.001*
Yes	105 (39.0)	98 (76.6)	203 (51.1)		
No	164 (61.0)	30 (23.4)	194 (48.9)		

EDs, eating disorders, ^*^significant; FE, Fisher’s Exact test.

## Discussion

Eating disorders remain a global health concern, especially among young individuals. A great challenge is the misconception that EDs are a lifestyle choice. In fact, EDs are serious and could be fatal conditions. EDs negatively affect eating behaviors, thoughts, and emotions. Medical studies can be highly stressful, and thereby, students’ mental health could be negatively impacted. Herein, we investigated the prevalence and associated possible risk factors of EDs among medical students. We also studied the response of medical students to a global stressor, COVID-19 pandemic, and reported the resultant effects in individuals at high risk for developing EDs.

In the present study, the distribution of females and males reflected the actual demographic data of the university. Most respondents were from preclinical years, specifically, years two and three. Alarmingly, among the 68.8% of the sample that reported struggling with mental health issues, only 18.6% claimed to have sought out help. A meta-analysis analyzing the barriers that medical students face in seeking help for mental health revealed that the most encountered barriers included fear of negative impact on residency and career opportunities, fear of confidentiality, breach and documentation on academic record and stigma and fear of shaming from peers (Fekih-Romdhane et al., 2022). These factors are especially prominent in a middle eastern context, as mental health remains a persistently stigmatized issue. Other factors include lack of perceived seriousness/normalization of symptoms, which is a common manifestation of denial especially in those struggling with eating disorders (Sewilam et al., 2015). A meta-analysis concluded that various barriers could limit seeking mental health care in Saudi Arabia, among which stigma was the most predominant barrier. Other barriers included cultural/religious beliefs, unawareness, confidentiality fears, lack of services, and cynicism. Among these barriers (Alhumaidan et al., 2024). Predominantly among medical student in Saudi Arabia, attitudinal and stigma-related barriers were the primary obstacles to seeking mental health assistance (Alsalman et al., 2024).

Unlike most comparable studies, the present study could not correlate gender to increased risk of EDs. Nevertheless, a similar finding was reported in previously (Naeimi et al., 2016). A possible explanation for this could be the limited sample size, or the special characters of the study cohort. It must be noted that the gender differences reported in the literature depended primarily on the ED under investigation. For example, females are more likely than males to experience AN or BN. On the other hand, females are either as likely as, or even less likely than, males to report BED (Striegel-Moore et al., 2009). Additionally, the use of EAT-26 as a case-finding tool is not well supported specially for measuring weight-related compensatory behaviors such as purging (McLean et al., 2023). Taken together, we can conclude that the lack of gender disparity in our hands does not contradict with the well-established correlation between gender and specific types of EDs.

Our results showed that 32.1% of participants classified as being at a higher risk of developing an eating disorder. Similar results were reported previously from different Middle Eastern countries. Studies on medical students from Saudi Arabia and Egypt reported that medical the 32–35% of medical students were at high risk of developing EDs (Abd El-Azeem Taha et al., 2018; Abo Ali, 2020; Ghamri et al., 2022). Controversial prevalence rates of EDs among medical students have also been reported. The prevalence of EDs among Palestinian female medical students was reported to be 28.6% (Saleh et al., 2018), while it was 22.8% among medical students in Pakistan with a female ratio of 7.3:1 (Memon et al., 2012). Several other reports showed much lower prevalence ratios among medical students. Liao et al. reported that the prevalence of EDs among Chinese medical university students was 2.46% (Liao et al., 2013). Studies on South Asian medical students showed a prevalence rate of 13% (Iyer and Shriraam, 2021). Additionally, the prevalence of EDs among medical students in Romania was reported to be 2.57% (Brumboiu et al., 2018), while it was estimated to be 3.24% among medical students in Puerto Rico (Reyes-Rodríguez et al., 2010). These reports point at a relatively higher risk of EDs among medical students in Bahrain as well as Bahrain’s neighboring countries, if compared to other countries in Asia, Europe, and South America. Interestingly, these observations highlight a notable difference in the risk of developing EDs among medical students according to geographical location, suggesting that different countries showcase different attitudes to eating and unique factors that contribute to an altered prevalence of distorted eating behaviors. The higher prevalence in the Arabian Gulf and neighboring Arab countries compared to the rest of the world may indicate an elevated dysfunction in eating attitudes in this region. In recent years, there has been an increase in bodily dissatisfaction, dieting tendencies and other forms of distorted eating in the Arab world (Melisse et al., 2024). This could be attributed to the rapid sociocultural changes that have occurred in the past 40 years because of western influences, widespread media usage, increased affluence, increased food options, high prevalence of obesity, and access to higher education (Melisse et al., 2020). Furthermore, sociocultural factors could influence the prevalence of EDs in the Middle East. Garrusi and Baneshi (2012) previously reported that in this part of the world, families prominently control the behavior of children, adolescents and even adults. Additionally, senior family members including grandparents interfere and shape the offspring lifestyles. The focus of Families on appearance and beauty has increased, promoting the sense of body dissatisfaction, and increasing the risk of EDs (Garrusi and Baneshi, 2012). This growing propensity to disordered eating of the population coupled with the stressful lifestyle medical students live put them at a constant risk of developing EDs (Abdulghani et al., 2011). Nevertheless, it is difficult to completely interpret the differences in prevalence rates of EDs among medical students worldwide due to inconsistent sample sizes of the different studies, as well as use of diverse screening tools.

In the present study, there was a significant association between a higher risk of developing an EDs and the study year. Students in the clinical years demonstrated a lower risk when compared to those in pre-clinical years. Our findings were in agreement with previously published reports (Ghamri et al., 2022). This correlation could be attributed to the major difficulties pre-clinical students face in navigating their way through medical school. In addition. Pre-clinical medical students must adjust to not only university life, but also to the demand and overwhelm of a medical curriculum. Particularly in the absence of a good coping method, students are less likely to overcome difficulties, resorting to ill-eating habits and/or obsession with their physical appearance (Liu et al., 2019).

Also, there was a significant association between the students’ living situation and the risk of developing ED in the present study. Those living alone or with their family had the greatest risk for disordered eating, while those living in private shared apartments and university dorms were at a lower risk. For instance, those living alone experience the struggles of social isolation which can lead to the feeling of disconnection and loneliness, both of which are risk factors for EDs (Cortés-García et al., 2022). The lack of emotional support from peers and family can make it harder for students to deal with the surrounding stressors of living abroad, increasing the risk of engagement in EDs as a coping mechanism. On the other hand, students living with their families may also encounter challenges that contribute to higher risk of EDs. Family dynamics, including high expectations, critical attitudes toward body image, and unhealthy eating patterns, can negatively impact an individual’s relationship with food and body image. Additionally, a family environment lacking open communication or emotional support can further increase the likelihood of developing EDs (Erriu et al., 2020). With such non-local students constituting 63.9% of the total high-risk proportion in our study, the higher risk among medical students living alone could be explained by several factors including familial separation, being deprived of the comforts and benefits of living at home, and perpetual stress of looking after themselves. An additional contributing factor is the lack of support of parents and family members, especially in the context of moving away (Liu et al., 2019). On the other hand, local students living among their families can suffer unique risks such as distress arising from conflict among members. In fact, poor familiar relationships were deemed a significant contributor to higher risk of developing EDs (Chang et al., 2015). Additionally, food being more readily available and socialized among Arab families could contribute to disordered eating as it takes away the friction of having to adhere to a budget or buying one’s own household groceries.

Another significant factor correlated with a high risk of developing EDs in the present study was having previously sought out help for mental health. This is not a surprising finding, as comorbid mental health conditions are well-established risk factors for developing EDs. Examples of such conditions include mood disorders, personality disorders, anxiety disorders and substance use disorders (Barakat et al., 2023). It has been found that psychiatric comorbidities were present in around 70% of people with established EDs, whether it preceded ED symptoms, occurred during the illness, or presented as a long-term complication (Juli et al., 2023).

In this study, the effect of COVID-19 pandemic, as a global stressor, on EDs. Major stressors negatively impact emotional wellbeing resulting in an increase in negative over positive emotions (Willroth et al., 2023). The present study provided a solid test examining eating habits trajectories over the COVID-19 pandemic. Among our study participants, 43.8% reported a deterioration in their eating habits during the pandemic. This percentage was particularly high among individuals in the lower risk eating disorder category, indicating a clear impact of the COVID-19 pandemic on individuals with subclinical eating disorders. Medical students, who already face various stressors, are more vulnerable to the negative effects of global stressors, which can lead to the development of a spectrum of eating disorders. The combination of quarantine measures and the academic burden of self-study can act as triggering factors for changes in the eating habits of medical students. According to the findings of Flaudias et al. (2020), there has been a notable rise in the number of young adults seeking treatment for eating disorders, both in inpatient and outpatient settings, since the onset of the COVID-19 pandemic. Furthermore, the study found that 44.1% of the participants expressed dissatisfaction with their food habits before and after the pandemic. This dissatisfaction indicates a negative impact on the participants’ relationship with food and a heightened risk of developing an eating disorder. The association between stress and EDs onset and symptom expression has been well documented. Stress may impact common neuronal circuitry that is involved in EDs (Hardaway et al., 2015). Research conducted in the United States (30, 31) indicated that university students experiencing food insecurity had a higher likelihood of testing positive for an eating disorder, particularly during the COVID-19 pandemic. Additionally, 55.7% of the participants reported using food as a means of comfort during the pandemic (Barry et al., 2021; Christensen et al., 2021). This finding highlights the complex interplay between emotional distress, coping mechanisms, social support, and routine disruption, which contribute to the increased reliance on food as a source of comfort during times of crisis. To further understand the complexity of the impact of a global stress on risk of EDs, it is crucial to explore the coping mechanisms individuals might had employed during the pandemic. Many turned to food for comfort during heightened stress, leading to emotional or binge eating to escape negative emotions. While this behavior may provide temporary relief, it often results in feelings of guilt and complicates one’s relationship with food. On the contrary, some individuals resorted to excessive exercise to regain control over their body image and cope with emotional distress. This pursuit of perfectionism could lead to unhealthy obsessions with fitness, potentially resulting in increased risk of EDs (Vuillier et al., 2021). Understanding these underlying mechanisms is crucial to develop interventions and support strategies to promote healthier coping mechanisms and emotional well-being during times of crisis which can further limit the potential of EDs. Furthermore, the psychosocial impact of the lockdown is not limited to the eating behavior of the medical students, it also interfered with the self-perception of their weight and body image. Moreover, more than 50% of the participants revealed an increased obsession with their weight and body image during the pandemic. Individuals who self-reported having an eating disorder during the pandemic exhibited challenges in controlling their eating behaviors, heightened concerns about body shape, increased thoughts about their body image, and engaged in more physical activity (Prati and Mancini, 2021). These findings underscore the significant relationship between the COVID-19 pandemic, as well as other major stressors, and the risk of EDs among medical students. Our results emphasized the need for support for individuals at risk for EDs during times of crisis and stress.

### Study limitations

Limitations include the self-reporting nature of the EAT-26 test, use of only one diagnostic tool, lack of clinical follow up data, and being conducted only in a single center. Additionally, the use of EAT-26 as a case-finding tool for measuring weight-related compensatory behaviors such as purging is not well supported. Although all the students in the sample were English speakers, yet the application of EAT-26 in an Arab population could be a limitation. EDs manifest differently in the west compared to the Arab world. Specifically, patients with EDs in the Arab world may express themselves somatically rather than psychiatrically.

## Conclusion

Medical students frequently suffer from disordered eating. Among the study group, living either with family or alone, being in early preclinical years, having mental health and/or psychiatric problems were the strongest indicators of EDs. This study draws attention to a health problem that medical students specifically and the public in general could undervalue. It is vital to introduce EDs prevention programs in medical schools to ultimately enhance the students’ quality of life and careers as future doctors. The most effective, and culturally appropriate, EDs prevention and treatment methods for medical students require more investigation.

## Data Availability

The original contributions presented in the study are included in the article/supplementary material, further inquiries can be directed to the corresponding author.
